# Association between Dupilumab and Conjunctivitis: A Systematic Review and Meta-Analysis of Randomized Controlled Trials

**DOI:** 10.3390/pharmaceutics15041031

**Published:** 2023-03-23

**Authors:** Tzu-Yi Lin, Ching-Ya Wang, Fang-Ying Wang, Eugene Yu-Chuan Kang, Yih-Shiou Hwang

**Affiliations:** 1 Department of Education, Chang Gung Memorial Hospital, Linkou Medical Center, Taoyuan 333, Taiwan; 2 School of Medicine, College of Medicine, Chang Gung University, Taoyuan 333, Taiwan; 3 Department of Dermatology, Chang Gung Memorial Hospital, Linkou Medical Center, Taoyuan 333, Taiwan; 4 Department of Ophthalmology, Chang Gung Memorial Hospital, Linkou Medical Center, Taoyuan 333, Taiwan; 5 Graduate Institute of Clinical Medical Sciences, College of Medicine, Chang Gung University, Taoyuan 333, Taiwan; 6 Department of Ophthalmology, Jen-Ai Hospital Dali Branch, Taichung 412, Taiwan; 7 Department of Ophthalmology, Xiamen Chang Gung Memorial Hospital, Xiamen 361000, China

**Keywords:** adverse event, atopic dermatitis, conjunctivitis, dupilumab, safety

## Abstract

Conjunctivitis is commonly reported in dupilumab users with atopic dermatitis (AD), and few studies have compared the risk of conjunctivitis among patients with different indications. This study aimed to investigate the association between dupilumab and conjunctivitis in various diseases. The protocol of this study was registered on PROSPERO (ID CRD42023396204). The electronic search of PubMed, Embase, Cochrane Library, and ClinicalTrials.gov was conducted for the period from their inception to January 2023. Only placebo-controlled, randomized controlled trials (RCTs) were included. The main outcome was the incidence of conjunctivitis during the study period. The subgroup analysis was performed for patients with AD and non-AD indications, which include asthma, chronic rhinosinusitis with nasal polyps, and eosinophilic esophagitis. In total, 23 RCTs involving 9153 patients were included for meta-analysis. Dupilumab users exhibited significantly higher risk of conjunctivitis (risk ratio [RR], 1.89; 95% confidence interval [CI], 1.34–2.67) than placebo users. Notably, significantly increased incidence of conjunctivitis was observed in the dupilumab group relative to the placebo group among patients with AD (RR, 2.43; 95% CI, 1.84–3.12) but not among patients with non-AD indications (RR, 0.71; 95% CI, 0.43–1.13). In conclusion, only dupilumab users with AD but not those with non-AD indications reported an elevated incidence of conjunctivitis.

## 1. Introduction

Dupilumab is the first human monoclonal antibody approved for inadequately controlled atopic dermatitis (AD) by the U.S. Food and Drug Administration and the European Medicines Agency in 2017 [1]. This agent blocks interleukin (IL)-4 receptor α, which is the shared subunit of IL-4 and IL-13 receptors and the key driver of various T-helper-2-mediated diseases [2,3]. Several randomized controlled trials (RCTs) have investigated the efficacy and safety of dupilumab to identify novel indications, such as asthma, chronic rhinosinusitis with nasal polyps (CRSwNP), and eosinophilic esophagitis (EE) [4,5,6,7,8,9,10,11]. Studies have reported that dupilumab is an effective and well tolerated therapy for moderate-to-severe AD, but ocular adverse events (e.g., blepharitis, conjunctivitis, keratitis, and dry eye disease) are commonly reported in patients who receive dupilumab [12,13,14]. Notably, conjunctivitis of all phenotypes and etiologies serves as the dominant ocular adverse event in the aforementioned RCTs of dupilumab [15].

The distinct pathophysiology of various indications may lead to different levels of conjunctivitis risk among dupilumab users. A higher incidence of conjunctivitis in the dupilumab group than in the placebo group was commonly reported in the RCTs of AD [16,17,18,19,20,21,22,23,24,25,26,27,28]. Despite this possible ocular adverse event, dupilumab revolutionized the management of moderate-to-severe AD among adults, adolescents, and children [29,30,31]. However, in the RCTs involving patients with non-AD indications, the dupilumab and placebo groups usually exhibited a similar risk of conjunctivitis [4,5,6,7,8,9,10,11]. Systematic reviews and meta-analyses have mostly focused on patients diagnosed with AD; by contrast, no study used the pooled data to compare the risk of conjunctivitis between dupilumab users with AD and those with non-AD indications [12,32]. To clarify the associations between dupilumab and conjunctivitis with different indications, we systematically reviewed all RCTs comparing dupilumab and placebo users in various diseases and synthesized the available data for meta-analysis. 

## 2. Materials and Methods

### 2.1. Search Strategy

The protocol of this study was registered on PROSPERO (ID CRD42023396204). Our systematic review and meta-analysis were conducted in accordance with the Preferred Reporting Items for Systematic Reviews and Meta-Analyses (PRISMA) guideline (Table S1). Two authors (Lin TY and Wang CY) independently searched the PubMed, Embase, Cochrane library, and ClinicalTrials.gov databases from their inception to January 2023. Specifically, titles and abstracts were screened to identify eligible studies. We used the keyword “dupilumab” to conduct an extensive search; the search strategy is presented in Table S2. Conjunctivitis is represented as an adverse event that occurs after the administration of dupilumab. The Medical Dictionary for Regulatory Activities preferred terms (MedDRA PTs) for conjunctivitis are conjunctivitis, allergic conjunctivitis, bacterial conjunctivitis, viral conjunctivitis, adenoviral conjunctivitis, and atopic keratoconjunctivitis.

### 2.2. Study Selection

Only RCTs that contained at least two arms, namely the dupilumab and placebo arms, were included in the present study. The studies containing treatment groups with concomitant usage of topical steroids were also included to expand the generalizability of this study. Articles were not limited to those in English, and no restriction was set for age, gender, or geography of the study population. If the same population was presented in multiple publications, only the most comprehensive data set was included in our study. The articles without a detailed abstract or full text were excluded from the present study. The references of the included studies were manually checked for potentially relevant articles. The full text of studies that were eligible for inclusion and those that were potentially eligible for inclusion was retrieved.

### 2.3. Data Extraction and Quality Assessment

The two authors (Lin TY and Wang CY) carefully read the full text, supplementary materials, and study protocol of each study. Information on the first author; the publication year; the type of RCT; the sample size; the participant characteristics; the indication, dosage, frequency, and drug survival associated with dupilumab; the incidence of conjunctivitis; and the treatment and follow-up periods were extracted using a predetermined form. The methodological quality of the included studies was evaluated using Cochrane Collaboration’s Risk of Bias tool, version 2. We assessed the risk of bias of each RCT based on five dimensions, namely randomization process, deviations from intended interventions, missing outcome data, measurement of outcome, and selection of reported results. Judgment was reported as “low risk,” “some concerns,” or “high risk.” The overall quality of evidence was evaluated using the Grading of Recommendations, Assessment, Development, and Evaluations (GRADE) system. Any discrepancy was resolved through discussions involving the corresponding authors. 

### 2.4. Data Synthesis and Analysis

Dichotomous outcomes are presented as risk ratios (RRs) with 95% confidence intervals (CIs). The heterogeneity among the studies was assessed with a chi-square test. The *I^2^* statistic was used to calculate the percentage of total variation among the studies. Different indications might suggest anticipated clinical heterogeneity, so the meta-analysis was performed with the random-effects model. A subgroup analysis was conducted for patients with AD and non-AD indications. A sensitivity analysis was performed by changing the applied methods, including Mantel–Haenszel and inverse variance methods. If an outcome was reported by >10 studies, publication bias was evaluated by visually inspecting funnel plots. A *p* value of <0.05 indicated statistical significance. All analyses were conducted using Review Manager (RevMan) software (version 5.4; The Nordic Cochrane Centre, The Cochrane Collaboration, 2014).

## 3. Results

### 3.1. Outcomes of the Literature Search and Characteristics of Included Studies

**Figure 1:**
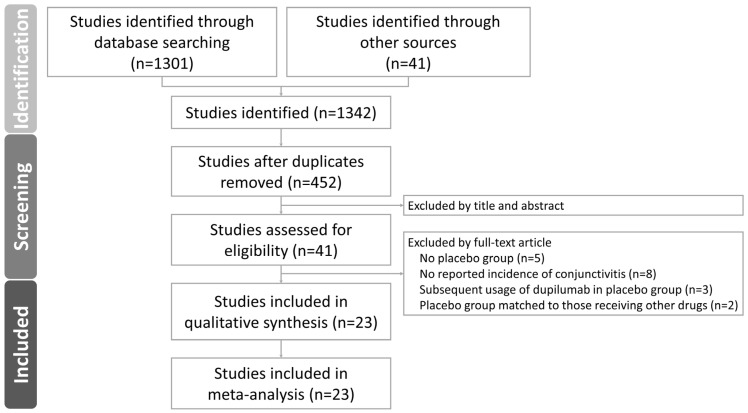
Flow diagram of study selection.

Throughout the search process, 1342 studies were retrieved, of which 890 studies were removed for duplication (Figure 1). Based on titles and abstracts, we excluded five studies with no placebo groups, eight studies with no reported incidence of conjunctivitis, three studies with subsequent use of dupilumab in the placebo group, and two studies with a placebo group that was matched to those receiving other drugs. Eventually, 21 articles covering 23 RCTs that reported usable results were included for analysis. These 23 RCTs were considered together for qualitative and quantitative analyses because they used similar protocols and examined similar pooled events. The characteristics of the included articles are presented in Table 1. These 23 RCTs examined various populations; specifically, 14 populations had AD, 4 populations had asthma, 3 populations had CRSwNP, and 2 populations had EE. Most of these RCTs were multinational trials, and only one was conducted only in Asia [26]. A summary of the risk of bias of the included studies is presented in Table S3. The blinding of the participants, investigators, and outcome assessors was considered adequate in most RCTs; outcome assessors were not masked in only one RCT [8]. Selective reporting was suspected in one RCT because its treatment period was longer than its follow-up period (treatment vs. follow-up was 52 weeks vs. 24 weeks) [8]. In Table S4, the overall quality of evidence for the association between dupilumab and conjunctivitis was low because of inconsistencies and imprecision. However, the overall quality of evidence obtained through our subgroup analyses of the patients with AD and non-AD indications was high. 

### 3.2. Conjunctivitis in Dupilumab versus Placebo Users

**Figure 2:**
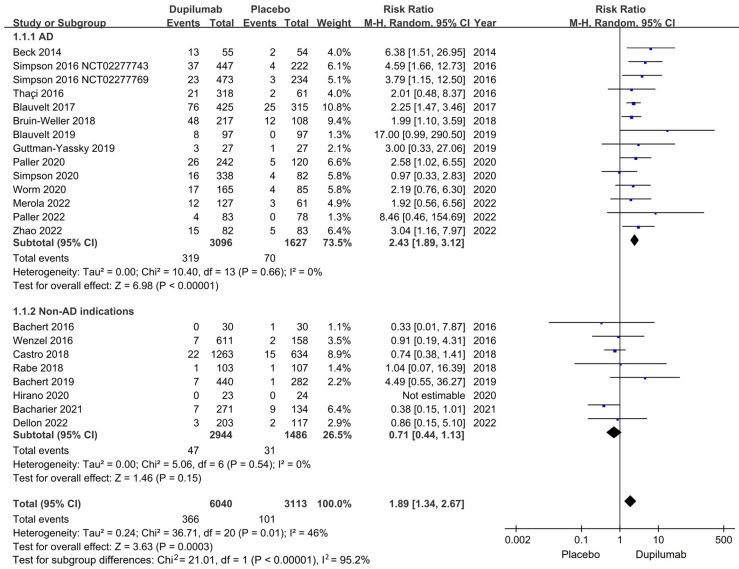
Forest plot of conjunctivitis in dupilumab versus placebo users. Abbreviation: AD, atopic dermatitis.

**Figure 3:**
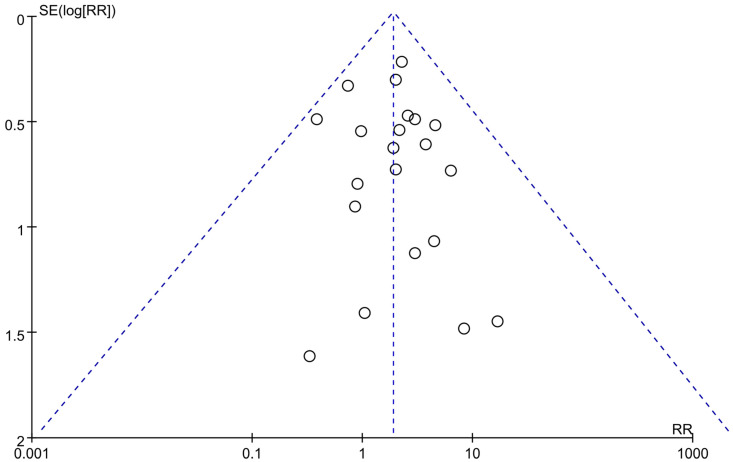
Funnel plot of conjunctivitis in dupilumab versus placebo users. Abbreviation: AD, atopic dermatitis.

In the 23 RCTs with 7797 patients, conjunctivitis was reported in 366 out of 6040 patients who received dupilumab and in 101 out of 3113 patients who received placebos. The dupilumab group exhibited a significantly higher incidence of conjunctivitis (RR, 1.89; 95% CI, 1.34–2.67; *I*^2^ = 46%, *p* = 0.01; Figure 2) relative to the placebo group. Among all the adult, adolescent, and child populations examined in the 23 RCTs, the risk of conjunctivitis was higher in dupilumab users than in placebo users. A sensitivity analysis revealed similar results for all methods (Table 2). No asymmetry was observed in the funnel plot generated in the present study (Figure 3). Most patients with conjunctivitis responded well to topical treatments. No patient discontinued their dupilumab therapy because of conjunctivitis.

### 3.3. Conjunctivitis in Dupilumab versus Placebo Users with AD and Non-AD indications

Based on the indications of dupilumab, we classified all RCTs into AD and non-AD groups for subgroup analysis. In the AD group, dupilumab users exhibited a significantly higher rate of conjunctivitis relative to placebo users (RR, 2.43; 95% CI, 1.89–3.12; *I*^2^ = 0%, *p* = 0.66; Figure 2). However, dupilumab and placebo users exhibited comparable rates of conjunctivitis in the non-AD group (RR, 0.71; 95% CI, 0.44–1.13; *I*^2^ = 0%, *p* = 0.54; Figure 2). A sensitivity analysis revealed similar results for all methods (Table 2).

## 4. Discussion

Our results indicated that dupilumab users exhibited a higher incidence of conjunctivitis than placebo users. Notably, an increased risk of conjunctivitis in the dupilumab group was observed merely among the patients with AD, which is consistent with the current evidence. However, this condition was not observed among the patients with non-AD indications.

In the present study, dupilumab users exhibited a significantly higher incidence of conjunctivitis relative to placebo users. A subgroup analysis revealed that a significantly higher rate of conjunctivitis was observed in the AD group but not in the non-AD group. The heterogeneity of all included RCTs was considerable; however, the heterogeneity of RCTs that examined AD and non-AD indications was adequate. The similar outcomes obtained through a sensitivity analysis confirmed the robustness of our results. A funnel plot asymmetry test did not reveal any publication bias in the present study. The GRADE system results indicated that the overall certainty of evidence for the association between dupilumab and conjunctivitis in the patients with AD and non-AD indications was high. Consistent with an early meta-analysis of four RCTs involving patients with AD, the dupilumab group of the present study exhibited a significantly higher risk of conjunctivitis (RR, 2.64; 95% CI, 1.79–3.89) relative to the placebo group of the present study [32]. Also, a recent study of multiple centers in Italy confirmed the great safety profile of dupilumab [33]. Conjunctivitis served as one of the most frequent adverse events (17.3%), and only a few adolescents discontinued dupilumab therapy due to conjunctivitis (1.6%). Similar to the information from the RCTs, dupilumab-associated conjunctivitis was usually mild in the real-world data.

This ocular adverse event could have been caused mainly by AD rather than dupilumab. AD can disrupt the immune-mediated response of the lymphoid tissue and impair the epithelial barrier in the conjunctiva [34,35]. Moreover, frequent rubbing due to itching can cause mechanical trauma to exaggerate the destruction of the ocular surface [36,37]. With the administration of dupilumab, the blockage of the signaling pathways of IL-4 and IL-13 further reduces the number of goblet cells of the conjunctiva in patients with AD [38,39]. The resulting decrease in mucin production can cause tear film instability and even conjunctivitis [40,41]. Increased eosinophilic factors in tears, an infestation of Demodex mites, and the increased activity of OX40 ligands have been proposed to explain the specific interplay between dupilumab and conjunctivitis in patients with AD; however, these hypotheses have yet to be proven [42,43,44]. Further investigations with ocular samples are required to characterize the molecular and cellular changes that occur before, during, and after dupilumab therapy.

Most patients who develop conjunctivitis lack ophthalmic referral for a full evaluation involving the use of standard tools. Notably, the differentiation between the flaring-up of AD-related conjunctivitis and dupilumab-associated conjunctivitis on the basis of clinical manifestations is a difficult task, and these two medical conditions share common pathogeneses. According to the study protocols of the included RCTs, ocular adverse events were generally diagnosed by dermatologists, allergists, or investigators [4,5,6,7,8,9,10,16,17,18,19,20,21,22,23,24,25,26]. In the included RCTs, blepharitis and keratitis, which were also common in patients with AD, were also challenging to distinguish from conjunctivitis. Furthermore, microbiologic tests were seldom conducted in these RCTs. Therefore, confirming whether a case of conjunctivitis was infectious, allergic, or idiopathic was difficult. The MedDRA PTs “conjunctivitis” (classified under the infections and infestations category) and “conjunctivitis allergic” (classified under the eye disorders category) were sometimes used ambiguously. A discrepancy in the assignment of conjunctivitis PTs might have limited the accuracy of the comparisons. In the present study, multiple MedDRA PTs were clustered to prevent fragmentation from occurring during signal detection.

Conjunctivitis causes various symptoms, including frequent itching and excessive discharge, which reduce a patient’s quality of life [45,46]. Moreover, the comorbidities of conjunctivitis (e.g., blepharitis, keratitis, and dry eye disease) can exaggerate the disruption of the ocular surface [47,48,49]. Insufficient protection and persistent inflammation can cause recurrent conjunctivitis [50,51]. This vicious cycle may lead to more severe diseases, such as corneal scar, corneal neovascularization, and recurrent corneal erosion syndrome, which require cornea transplantation to prevent blindness [52,53,54]. Consequently, timely identification and intervention are essential for dupilumab users with AD because of their high risk of conjunctivitis. A consensus regarding the optimal approach for managing the conjunctivitis of dupilumab users has yet to be reached. A clear protocol is required for patients with AD who intend to use dupilumab. All patients who develop conjunctivitis should be referred to ophthalmologists for a full evaluation. Thereafter, therapeutic strategies can be individualized for each patient based on the type and severity of their conjunctivitis, concomitant medication use, and the presence of comorbidities. Administering timely and effective treatments for conjunctivitis can increase the quality of life of patients undergoing dupilumab therapy.

The present systematic review and meta-analysis of RCTs still has some limitations. First, an ophthalmic referral was not necessary in the study protocols of most of the included RCTs. The lack of a thorough evaluation may lead to the underdiagnosis or overdiagnosis of conjunctivitis. Nevertheless, this relative effect should be unbiased because no difference was detected between dupilumab and placebo users. Second, the duration and treatment of conjunctivitis could not be analyzed in this systematic review and meta-analysis. Most included RCTs merely reported the incidence of adverse events and did not present the follow-up data. Further studies were required to acquire the information to deal with dupilumab-associated conjunctivitis. 

Second, the association between ocular history and the incidence of conjunctivitis after dupilumab therapy could not be investigated. High prevalence of ocular surface diseases was reported in the patients with moderate-to-severe AD before dupilumab therapy [55,56]. Moreover, the patients with ocular surface diseases at baseline tended to develop conjunctivitis with higher severity than those without [15]. However, ophthalmic evaluation at baseline was not necessarily performed in most RCTs, and not all patients provided their ocular history. Also, the differentiation between flaring-up of AD-related or dupilumab-associated adverse events could be challenging due to the lack of information. 

Third, awareness bias was identified based on the increasing rate of conjunctivitis in the placebo groups in various included RCTs over time. The increase in physician attention and patient reporting increased the prevalence at baseline and the incidence during the study periods [57]. Additionally, conjunctivitis, which is a known ocular adverse event, may prevent investigators from including patients with severe ocular diseases. Furthermore, recall bias could have occurred when ocular symptoms were misinterpreted by patients or investigators [58]. Finally, the CRSwNP and EE populations examined by the included RCTs were small because the indications pertaining to these diseases are still being developed. In further analyses, data from ongoing and future studies should be incorporated.

## 5. Conclusions

Dupilumab users exhibited a higher incidence of conjunctivitis relative to placebo users. Notably, an increased rate of conjunctivitis in the dupilumab group was detected in the patients with AD, which is consistent with the current evidence. However, this condition was not observed in the patients with non-AD indications. Further studies are required to clarify the pathogenesis of conjunctivitis in dupilumab users with AD and to develop standardized guidelines for managing and even preventing conjunctivitis.

## Figures and Tables

**Table 1:** Characteristics of included studies.

Publication (RCT Identifier)	Indication of Dupilumab	Type of RCT	Number of Patients	Age *	Gender ^#^	Drug Survival of Dupilumab	Incidence of Conjunctivitis	Follow-Up Time of AEs
Beck 2014 (NCT01548404)	Atopic dermatitis	Two-arm, triple-blind, placebo-controlled RCT	Dupilumab 300mg QW (*n* = 55) vs. Placebo QW (*n* = 54) for 12 weeks	Adult 36.5 (11.69)	46.8%/53.2%	74.55% (41/55)	23.64% (13/55) dupilumab vs. 3.70% (2/54) placebo	28 weeks
Simpson 2016 (NCT02277743) (NCT02277769)	Atopic dermatitis	Three-arm, triple-blind, placebo-controlled RCT	Dupilumab 300mg QW (*n* = 223) vs. Dupilumab 300mg Q2W (*n* = 224) vs. Placebo QW (*n* = 224) for 16 weeks	Adult 39.5 (14.31)	41.9%/58.1%	90.60% (405/447)	6.52% (60/920) dupilumab vs. 1.54% (7/456) placebo	28 weeks
	Atopic dermatitis	Three-arm, triple-blind, placebo-controlled RCT	Dupilumab 300mg QW (*n* = 239) vs. Dupilumab 300mg Q2W (*n* = 233) vs. Placebo QW (*n* = 236) for 15 weeks	Adult 37.1 (14.17)	42.4%/57.6%	93.45% (441/473)		28 weeks
Thaçi 2016 (NCT01859988)	Atopic dermatitis	Six-arm, quadruple-blind, placebo-controlled RCT	Dupilumab 300 mg QW (*n* = 63) vs. Dupilumab 300 mg Q2W (*n* = 64) vs. Dupilumab 200 mg Q2W (*n* = 62) vs. Dupilumab 300 mg Q4W (*n* = 65) vs. Dupilumab 100 mg Q4W (*n* = 65) vs. Placebo QW (*n* = 61) for 15 weeks	Adult 37.0 (12.06)	38.3%/61.7%	73.90% (235/318)	6.60% (21/318) dupilumab vs. 3.28% (2/61) placebo	32 weeks
Blauvelt 2017 (NCT02260986)	Atopic dermatitis	Three-arm, triple-blind, placebo-controlled RCT	Dupilumab 300mg QW (*n* = 319) vs. Dupilumab 300mg Q2W (*n* = 106) vs. Placebo QW (*n* = 315) for 51 weeks	Adult 37.1 (13.46)	39.7%/60.3%	91.75% (371/425)	17.88% (76/425) dupilumab vs. 7.94% (25/315) placebo	52 weeks
Bruin-Weller 2018 (NCT02755649)	Atopic dermatitis	Three-arm, quadruple-blind, placebo-controlled RCT	Dupilumab 300mg QW +TCS (*n* = 110) vs. Dupilumab 300mg Q2W+TCS (*n* = 107) vs. Placebo QW +TCS (*n* = 108) for 16 weeks	Adult 38.4 (13.13)	38.8%/61.2%	99.08% (215/217)	22.12% (48/217) dupilumab vs. 11.11% (12/108) placebo	28 weeks
Blauvelt 2019 (NCT02210780)	Atopic dermatitis	Two-arm, triple-blind, placebo-controlled RCT	Dupilumab 300mg QW (*n* = 97) vs. Placebo QW (*n* = 97) for 15 weeks	Adult 39.6 (13.77)	51.0%/49.0%	91.75% (89/97)	8.25% (8/97) dupilumab vs. 0.00% (0/97) placebo	32 weeks
Guttman-Yassky 2019 (NCT01979016)	Atopic dermatitis	Two-arm, quadruple-blind, placebo-controlled RCT	Dupilumab 200mg QW (*n* = 27) vs. Placebo QW (*n* = 27) for 15 weeks	Adult 41.3 (14.81)	44.4%/55.6%	96.30% (26/27)	11.11% (3/27) dupilumab vs. 3.70% (1/27) placebo	32 weeks
Paller 2020 (NCT03345914}	Atopic dermatitis	Three-arm, quadruple-blind, placebo-controlled RCT	Dupilumab 300mg Q4W +TCS (*n* = 120) vs. Dupilumab 300/100mg Q2W+TCS (*n* = 122) vs. Placebo QW +TCS (*n* = 120) for 16 weeks	Child 8.5 (1.72)	50.1%/49.9%	97.13% (237/244)	10.74% (26/242) dupilumab vs. 4.17% (5/120) placebo	28 weeks
Worm 2020 (NCT02395133)	Atopic dermatitis	Four-arm, triple-blind, placebo-controlled RCT	Dupilumab 300mg QW/Q2W (*n* = 169) vs. Dupilumab 300mg Q4W (*n* = 86) vs. Dupilumab 300mg Q8W (*n* = 84) vs. Placebo QW (*n*=83) for 36 weeks	Adult 38.2 (14.46)	46.2%/53.8%	90.27% (306/339)	4.73% (16/338) dupilumab vs. 4.88% (4/82) placebo	36 weeks
Simpson 2020 (NCT03054428)	Atopic dermatitis	Three-arm, quadruple-blind, placebo-controlled RCT	Dupilumab 300/200mg Q2W (*n* = 82) vs. Dupilumab 300mg Q4W (*n* = 84) vs. Placebo Q2W (*n* = 85) for 16 weeks	Adolescent 14.5 (1.70)	41.0%/59.0%	96.39% (160/166)	10.30% (17/165) dupilumab vs. 4.71% (4/85) placebo	28 weeks
Merola 2022 (NCT04033367)	Atopic dermatitis	Two-arm, quadruple-blind, placebo-controlled RCT	Dupilumab 300mg Q2W (*n* = 127) vs. Placebo Q2W (*n* = 61) for 12 weeks	Adult 35.7 (14.89)	51.6%/48.4%	96.06% (122/127)	9.45% (12/127) dupilumab vs. 4.92% (3/61) placebo	12 weeks
Paller 2022 (NCT03346434)	Atopic dermatitis	Two-arm, quadruple-blind, placebo-controlled RCT	Dupilumab 400/200mg Q4W + TCS (*n* = 83) vs. Placebo Q2W + TCS (*n* = 79) for 12 weeks	Child 4.0 (0.8)	61.1%/38.9%	100.00% (83/83)	4.82% (4/83) dupilumab vs. 0.00% (0/78) placebo	28 weeks
Zhao 2022 (NCT03912259)	Atopic dermatitis	Two-arm, quadruple-blind, placebo-controlled RCT	Dupilumab 300mg Q2W (*n* = 82) vs. Placebo Q2W (*n*=83) for 16 weeks	Adult 30.6 (11.49)	28.5%/71.5%	92.68% (76/82)	18.29% (15/82) dupilumab vs. 6.02% (5/83) placebo	28 weeks
Wenzel 2016 (NCT01854047)	Asthma	Five-arm, triple-blind, placebo-controlled RCT	Dupilumab 300mg Q2W (*n* = 154) vs. Dupilumab 200mg Q2W (*n* = 157) vs. Dupilumab 300mg Q4W (*n* = 150) vs. Dupilumab 100mg Q4W (*n* = 157) vs. Placebo Q2W (*n* = 158) for 22 weeks	Adult 48.6 (13.00)	63.1%/36.9%	92.14% (563/611)	1.15% (7/611) dupilumab vs. 1.27% (2/158) placebo	40 weeks
Castro 2018 (NCT02414854)	Asthma	Four-arm, triple-blind, placebo-controlled RCT	Dupilumab 300mg Q2W (*n* = 633) vs. Dupilumab 200mg Q2W (*n* = 631) vs. Placebo 300mg Q2W (*n* = 321) vs. Placebo 200mg Q2W (*n* = 317) for 50 weeks	Adult 47.9 (15.30)	62.9%/37.1%	92.41% (1168/1264)	1.74% (22/1263) dupilumab vs. 2.37% (15/634) placebo	64 weeks
Rabe 2018 (NCT02528214)	Asthma	Two-arm, triple-blind, placebo-controlled RCT	Dupilumab 300mg Q2W (*n* = 103) vs. Placebo Q2W (*n* = 107) for 24 weeks	Adult 51.3 (12.60)	60.5%/39.5%	97.09% (100/103)	0.97% (1/103) dupilumab vs. 0.93% (1/107) placebo	36 weeks
Bacharier 2021 (NCT02948959)	Asthma	Two-arm, triple-blind, placebo-controlled RCT	Dupilumab 200/100mg Q2W (*n* = 273) vs. Placebo Q2W (*n*=135) for 52 weeks	Child 8.9 (1.6)	35.8%/64.2%	90.84% (248/273)	2.58% (7/271) dupilumab vs. 6.72% (9/134) placebo	64 weeks
Bachert 2016 (NCT01920893)	CRSwNP	Two-arm, triple-blind, placebo-controlled RCT	Dupilumab 300mg QW (*n* = 30) vs. Placebo QW (*n* = 30) for 15 weeks	Adult 48.4 (9.40)	43.3%/56.7%	93.33% (28/30)	0.00% (0/30) dupilumab vs. 3.33% (1/30) placebo	32 weeks
Bachert 2019 (NCT02898454) (NCT02912468)	CRSwNP	Three-arm, quadruple-blind, placebo-controlled RCT	Dupilumab 300mg Q2W (*n* = 150) vs. Dupilumab 300mg Q2W then Q4W (*n* = 145) vs. Placebo Q2W (*n* = 153) for 52 weeks	Adult 51.95 (12.45)	37.7%/62.3%	96.27% (284/295)	1.59% (7/440) dupilumab vs. 0.35% (1/282) placebo	24 weeks
	CRSwNP	Two-arm, double-blind, placebo-controlled RCT	Dupilumab 300mg Q2W (*n* = 143) vs. Placebo Q2W (*n* = 133) for 24 weeks	Adult 50.49 (13.39)	42.8%/57.2%	96.50% (138/143)		24 weeks
Hirano 2020 (NCT02379052)	EE	Two-arm, triple-blind, placebo-controlled RCT	Dupilumab 300mg QW (*n* = 23) vs. Placebo QW (*n* = 24) for 12 weeks	Adult 34.7 (10.94)	51.1%/48.9%	78.26% (18/23)	0.00% (0/23) dupilumab vs. 0.00% (0/24) placebo	28 weeks
Dellon 2020 (NCT03633617)	EE	Five-arm, quadruple-blind, placebo-controlled RCT	Part A Dupilumab 300mg QW (*n* = 42) vs. Placebo QW (*n* = 39) for 24 weeks Part B Dupilumab 300mg QW (*n* = 80) vs. Dupilumab 300mg Q2W (*n* = 81) vs. Placebo QW (*n* = 79) for 24 weeks	Adult 28.1 (13.12)	63.7%/36.3%	94.09% (191/203)	1.48% (3/203) dupilumab vs. 1.71% (2/117) placebo	24 weeks

Abbreviations: AE, adverse event; CRSwNP, chronic rhinosinusitis with nasal polyps; EE, eosinophilic esophagitis; RCT, randomized controlled trial. * Data were presented as means (standard deviations, SDs). **^#^** Data were presented as proportion of female to male patients.

**Table 2:** Sensitivity analysis through various methods for included studies *.

Methods	Models	Odds Ratio (95%Cl)	Relative Risk (95%Cl)
**Conjunctivitis in dupilumab versus placebo users**			
Mantel–Haenszel	Random effects	2.01 (1.38–2.93)	1.89 (1.34–2.67)
Inverse variance	Random effects	2.01 (1.39–2.92)	1.89 (1.34–2.66)
**Conjunctivitis in dupilumab versus placebo users with AD**			
Mantel–Haenszel	Random effects	2.71 (2.06–3.55)	2.43 (1.89–3.12)
Inverse variance	Random effects	2.71 (2.06–3.55)	2.43(1.85–3.61)
**Conjunctivitis in dupilumab versus placebo users with non-AD indications**			
Mantel–Haenszel	Random effects	0.71 (0.44–1.14)	0.71 (0.44–1.13)
Inverse variance	Random effects	0.70 (0.42–1.14)	0.71 (0.44–1.13)

Abbreviations: AD, atopic dermatitis; CI, confidence interval; N/A, not applicable. * Studies with no events in both groups were excluded.

## Data Availability

The datasets generated during and analyzed during the current study are available from the corresponding author on reasonable request.

## Supplementary Materials

The following supporting information can be downloaded at https://www.mdpi.com/article/10.3390/pharmaceutics15041031/s1: Table S1: Preferred Reporting Items for Systematic Reviews and Meta-Analyses (PRISMA) of study; Table S2: Search strategy of study; Table S3: Summary of authors’ judgement of risk of bias of included studies; Table S4: Grading of recommendations assessment, development, and evaluation (GRADE) evidence table for conjunctivitis in patients receiving dupilumab versus placebo.
