# Supplementary material for: Association between Dupilumab and Conjunctivitis: A Systematic Review and Meta-Analysis of Randomized Controlled Trials
Source: Pharmaceutics. 2023 Mar 23;15(4):1031. doi: 10.3390/pharmaceutics15041031 (PMC10145140; doi:10.3390/pharmaceutics15041031)
Supplement: Supplementary file 1 [file pharmaceutics-15-01031-s001.zip › 230218_Table S2.pdf]

Table S2 Search strategy of study

**PubMed**	("dupilumab"[Supplementary Concept] OR "dupilumab"[All Fields] OR "dupixent"[All Fields] OR "sar231893"[All Fields] OR "sar 231893"[All Fields] OR "regn668"[All Fields] OR "regn 668"[All Fields])
**Embase**	'dupilumab'/exp OR dupilumab OR Dupixent OR sar231893 OR 'sar 231893' OR regn668 OR 'regn 668'
**Cochrane library**	dupilumab OR Dupixent OR SAR231893 OR SAR-231893 OR REGN668 OR REGN-668
**ClinicalTrial.gov**	dupilumab OR Dupixent OR SAR231893 OR SAR-231893 OR REGN668 OR REGN-668
