# Supplementary material for: Association between Dupilumab and Conjunctivitis: A Systematic Review and Meta-Analysis of Randomized Controlled Trials
Source: Pharmaceutics. 2023 Mar 23;15(4):1031. doi: 10.3390/pharmaceutics15041031 (PMC10145140; doi:10.3390/pharmaceutics15041031)
Supplement: Supplementary file 1 [file pharmaceutics-15-01031-s001.zip › 230218_Table S3.pdf]

Table S3 Summary of authors’ judgement of risk of bias of included studies.

Study	Trial	D1	D2	D3	D4	D5	Overall
Beck 2014	NCT01548404	🟢	🟢	🟢	🟢	🟢	🟢
Simpson 2016	NCT02277743	🟢	🟢	🟢	🟢	🟢	🟢
	NCT02277769	🟢	🟢	🟢	🟢	🟢	🟢
Thaçi 2016	NCT01859988	🟢	🟢	🟢	🟢	🟢	🟢
Blauvelt 2017	NCT02260986	🟢	🟢	🟢	🟢	🟢	🟢
Bruin-Weller 2018	NCT02755649	🟢	🟢	🟢	🟢	🟢	🟢
Blauvelt 2019	NCT02210780	🟢	🟢	🟢	🟢	🟢	🟢
Guttman-Yassky 2019	NCT01979016	🟢	🟢	🟢	🟢	🟢	🟢
Paller 2020	NCT03345914	🟢	🟢	🟢	🟢	🟢	🟢
Worm 2020	NCT02395133	🟢	🟢	🟢	🟢	🟢	🟢
Simpson 2020	NCT03054428	🟢	🟢	🟢	🟢	🟢	🟢
Merola 2022	NCT04033367	🟢	🟢	🟢	🟢	🟢	🟢
Paller 2022	NCT03346434	🟢	🟢	🟢	🟢	🟢	🟢
Zhao 2022	NCT03912259	🟢	🟢	🟢	🟢	🟢	🟢
Bachert 2016	NCT01920893	🟢	🟢	🟢	🟢	🟢	🟢
Wenzel 2016	NCT01854047	🟢	🟢	🟢	🟢	🟢	🟢
Castro 2018	NCT02414854	🟢	🟢	🟢	🟢	🟢	🟢
Rabe 2018	NCT02528214	🟢	🟢	🟢	🟢	🟢	🟢
Bachert 2019	NCT02898454	🟢	🟢	🟢	🟢	🔴	🔴
	NCT02912468	🟢	🟢	🟢	🟡	🟢	🟡
Hirano 2020	NCT02379052	🟢	🟢	🟢	🟢	🟢	🟢
Bacharier 2021	NCT02948959	🟢	🟢	🟢	🟢	🟢	🟢
Dellon 2020	NCT03633617	🟢	🟢	🟢	🟢	🟢	🟢

🟢 Low risk

🟡 Some concerns

🔴 High risk

D1 Randomization process

D2 Deviations from intended interventions

D3 Missing outcome data

D4 Measurement of outcomes

D5 Selection of reported results
