# Supplementary material for: Association between Dupilumab and Conjunctivitis: A Systematic Review and Meta-Analysis of Randomized Controlled Trials
Source: Pharmaceutics. 2023 Mar 23;15(4):1031. doi: 10.3390/pharmaceutics15041031 (PMC10145140; doi:10.3390/pharmaceutics15041031)
Supplement: Supplementary file 1 [file pharmaceutics-15-01031-s001.zip › 230218_Table S4.pdf]

Table S4 Grading of recommendations assessment, development and evaluation (GRADE) evidence table for conjunctivitis in patients receiving dupilumab versus placebo.

Certainty assessment							Event rate		Effect		Certainty	Importance
Number of studies	Study design	Risk of bias	Inconsistency	Indirectness	Imprecision	Other considerations	Dupilumab	Placebo	Relative (95% CI)	Absolute (95% CI)		
Conjunctivitis in patients receiving dupilumab versus placebo												
23	RCTs	not serious	serious[a]	not serious	serious[b]	none	366/6040 (6.1%)	101/3113 (3.2%)	RR 1.89 (1.34 to 2.67)	29 more per 1,000 (from 11 more to 54 more)	⊕⊕○○ Low	Critical
Conjunctivitis in patients with AD receiving dupilumab versus placebo												
14	RCTs	not serious	not serious	not serious	not serious	none	319/3096 (10.3%)	70/1627 (4.3%)	RR 2.43 (1.89 to 3.12)	63 more per 1,000 (from 38 more to 91 more)	⊕⊕⊕⊕ High	Critical
Conjunctivitis in patients with non-AD indications receiving dupilumab versus placebo												
9	RCTs	not serious	not serious	not serious	not serious	none	47/2944 (1.6%)	31/1486 (2.1%)	RR 0.71 (0.44 to 1.13)	6 fewer per 1,000 (from 12 fewer to 3 more)	⊕⊕⊕⊕ High	Critical

Abbreviations: CI, confidence interval; RCT, randomized controlled trial; RR, risk ratio.

[a] I^2 of 59% and *P*-value of 0.002 indicate heterogeneity of included studies.

[b] Confidence interval includes both appreciable benefit and harm.
